# Precipitation behavior of Al_*x*_CoCrFeNi high entropy alloys under ion irradiation

**DOI:** 10.1038/srep32146

**Published:** 2016-08-26

**Authors:** Tengfei Yang, Songqin Xia, Shi Liu, Chenxu Wang, Shaoshuai Liu, Yuan Fang, Yong Zhang, Jianming Xue, Sha Yan, Yugang Wang

**Affiliations:** 1 State Key Laboratory of Nuclear Physics and Technology, Center for Applied Physics and Technology, Peking University, Beijing 100871, People’s Republic of China; 2 State Key Laboratory for Advanced Metals and Materials, University of Science and Technology Beijing, Beijing 100083, China

## Abstract

Materials performance is central to the satisfactory operation of current and future nuclear energy systems due to the severe irradiation environment in reactors. Searching for structural materials with excellent irradiation tolerance is crucial for developing the next generation nuclear reactors. Here, we report the irradiation responses of a novel multi-component alloy system, high entropy alloy (HEA) Al_*x*_CoCrFeNi (*x* = 0.1, 0.75 and 1.5), focusing on their precipitation behavior. It is found that the single phase system, Al_0.1_CoCrFeNi, exhibits a great phase stability against ion irradiation. No precipitate is observed even at the highest fluence. In contrast, numerous coherent precipitates are present in both multi-phase HEAs. Based on the irradiation-induced/enhanced precipitation theory, the excellent structural stability against precipitation of Al_0.1_CoCrFeNi is attributed to the high configurational entropy and low atomic diffusion, which reduces the thermodynamic driving force and kinetically restrains the formation of precipitate, respectively. For the multiphase HEAs, the phase separations and formation of ordered phases reduce the system configurational entropy, resulting in the similar precipitation behavior with corresponding binary or ternary conventional alloys. This study demonstrates the structural stability of single-phase HEAs under irradiation and provides important implications for searching for HEAs with higher irradiation tolerance.

Structural materials used in nuclear reactors must maintain both mechanical performance and dimensional stability under irradiation environments^1^. The next generation of nuclear reactor will be more efficient and economical and produce less radioactive waste, this requires the structural materials can withstand severer environment, such as higher temperatures and irradiation doses. Therefore, advanced nuclear reactor designs call for dramatic progress in materials, and numerous new materials were investigated for using in advanced nuclear reactors, such as oxide dispersion steel (ODS)^2^, bulk metallic glass (BMG)^3^ and bulk nanolayered (NL) composites^4^.

Metallic alloys are generally based on one or two major elements. Various alloying elements are added to modify the alloy properties. In 2004, Yeh *et al.*^5^ proposed a new class of alloys referred to as “high-entropy alloys” (HEAs), which are defined as consisting of at least five principal elements in equiatomic or near equiatomic concentrations. The high entropy of mixing benefits the formation of solid-solution phases with simple structures and suppresses the formation of numerous intermetallic phases, avoiding the disadvantages of conventional multicomponent alloys. Many HEA systems have been explored and their mechanical properties were widely investigated^6^. It is found HEAs can exhibit high hardness^5^, encouraging fatigue resistance^7^, wear resistance^8^ and excellent low temperature fracture-resistant^9^. Specially, due to the high configurational entropy of mixing of HEAs, the reduction of system free energy significantly increases with temperature increasing, resulting in the great thermodynamical high-temperature stability and suggesting a promising potential in the high-temperature applications^10^. Many HEA systems with very good mechanical properties and thermal stability at high temperatures have been explored^11^,^12^.

Due to the good mechanical properties and structural stability, HEAs have attracted much attention recently for using as nuclear materials^13^. Despite HEAs exhibiting attractive mechanical properties, their structure and composition may be changed under irradiation, which will significantly degrade their performances. Therefore, the structure and composition stabilities of HEAs under irradiation are crucial for their applications as nuclear materials and a deep understanding about the irradiation responses of HEAs can provide important implications for searching for and preparing HEAs with higher irradiation tolerance.

In the present study, the structure and phase stabilities of HEAs under ion irradiation are studied. A well studied HEA system, Al_*x*_CoCrFeNi, is chosen as a model system. Three different Al contents, *x* = 0.1, 0.75 and 1.5, of which structures are single fcc, fcc plus ordered bcc (B2) and B2 plus disordered bcc (A2), respectively, are employed^14^. The microstructures of the three HEAs irradiated with Au ions are studied and compared with each other. Due to the multiple alloying elements, precipitation and phase separation could occur, which will appreciably change the structure and composition of HEAs and result in the degradation of mechanical properties, such as embrittlement^15^,^16^,^17^. Therefore, the comparison of irradiation responses is focused on precipitation behavior. The experiment results are also compared with the conventional alloys and discussed based on theory of irradiation-induced/enhanced precipitation which is widely used in immiscible binary systems. The aim of current study is to investigate the structural stability of HEAs under ion irradiation and explore the microstructure and chemical composition dependences of irradiation tolerances of HEAs.

## Results

### Irradiation-induced structural evolutions in Al_*x*_CoCrFeNi HEAs

**Figure 1:**
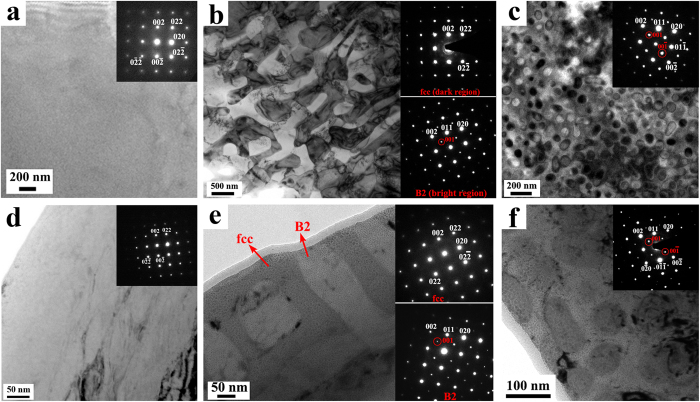
Bright field (BF) TEM images and corresponding selected area electron diffraction (SAED) patterns of virgin (**a~c**) and as-irradiated (**d~f**) Al_*x*_CoCrFeNi HEAs. The ion irradiation was performed with 3 MeV Au ions at 1 × 10^16^ cm^−2^. (**a**,**c**) *x* = 0.1, (**b,e**) *x* = 0.75, (**c,f**) *x* = 1.5. Figure 1(**d**) is taken with electron beam far away from [100] zone axis to avoid the interference of ion irradiation-induced defects to the observation of precipitates (SAED is still taken along [100] zone axis), while the other images are taken with electron beam along [100] zone axis.

Figure 1 shows the bright field (BF) TEM images and corresponding selected area electron diffraction (SAED) patterns of virgin (a~c) and as-irradiated (d~f) Al_*x*_CoCrFeNi HEAs. Only a single fcc phase was found in virgin Al_0.1_CoCrFeNi alloy, and no other phase or nano-scale precipitate can be found, as shown in Fig. 1(a). Al_0.75_CoCrFeNi displays an alternating interconnected two phase microstructure, consisting of B2 (ordered bcc, bright regions) and fcc phases (dark regions), respectively, as indicated by SAED patterns in Fig. 1(b). For *x* = 1.5, TEM microstructural characterization of the as-fabricated alloy (Fig. 1(c)) revealed a high density of spherical precipitates with average diameter of ~80 nm distributed throughout the matrix. TEM dark field (DF) images (not shown here) indicated that the spherical precipitates have A2 structure (disordered bcc) and the matrix is B2 structure. TEM-EDX characterization reveals that B2 phases in both Al_0.75_CoCrFeNi and Al_1.5_CoCrFeNi are enriched in Al and Ni, the fcc phases and A2 phase are enriched in Fe, Cr, Co and Fe, Cr, respectively. The EDX results are summarized in Table 1.

**Figure 2:**
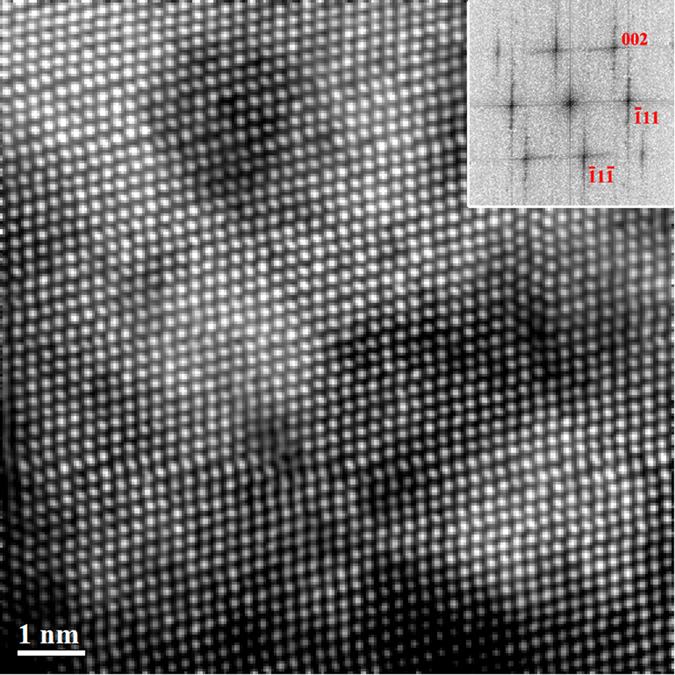
HAADF image and corresponding FFT of the precipitates in the fcc phase of as-irradiated Al_0.75_CoCrFeNi (1 × 10^16^ cm^−2^, ~40 dpa), showing the coherent relationship between precipitates (brighter contrast) and matrix (darker contrast).

Figure 1(d~f) show the BF images and SAED patterns of Al_*x*_CoCrFeNi irradiated with 3 MeV Au ions at 1 × 10^16^ cm^−2^. Compared with the SAED patterns of as-fabricated Al_*x*_CoCrFeNi alloys, no significant variation can be found in that of as-irradiated samples, suggesting the initial microstructures should be essentially preserved for the three as-irradiated alloys. The chemical compositions of different phases in the three as-irradiated HEAs (Table 1) are also remained as compared with the virgin samples. The most apparent microstructural characteristic of the as-irradiated HEAs is the precipitation. No precipitate can be observed In the BF image of Al_0.1_CoCrFeNi (Fig. 1(d)). In contrast, numerous nano-scale precipitates can be found in both as-irradiated Al_0.75_CoCrFeNi (Fig. 1(e)) and Al_1.5_CoCrFeNi (Fig. 1(f)). These precipitates are uniformly distributed in different phases, suggesting the homogeneous nucleation mechanism. Moreover, SAED patterns indicate that these precipitates are coherent with corresponding matrix. A representative high angle annular dark field (HAADF) image and corresponding Fast Fourier Transform (FFT) of the precipitates in the fcc phase of as-irradiated Al_0.75_CoCrFeNi is given in Fig. 2, which clearly shows the coherent relationship. These coherent precipitates can easily coalesce with each other under continuous growth. The elongation of transformed spots in FFT results from irradiation-induced lattice distortion, which can be also observed in HAADF image. It should be noted that the nanoscale precipitates can be also observed at a fluence of 5 × 10^15^ cm^−2^, and it will be further discussed. Furthermore, ion irradiation-induced structural damage can be also observed in the three studied HEAs, as shown in Supplementary Fig. S1. However, in current study we focus on the precipitation behavior of different HEAs, the comprehensive study of the irradiation-induced defects and structural damage in the three HEAs will be published in future.

### Comparison of nanoscale precipitation in as-irradiated Al_*x*_CoCrFeNi alloys

**Figure 3:**
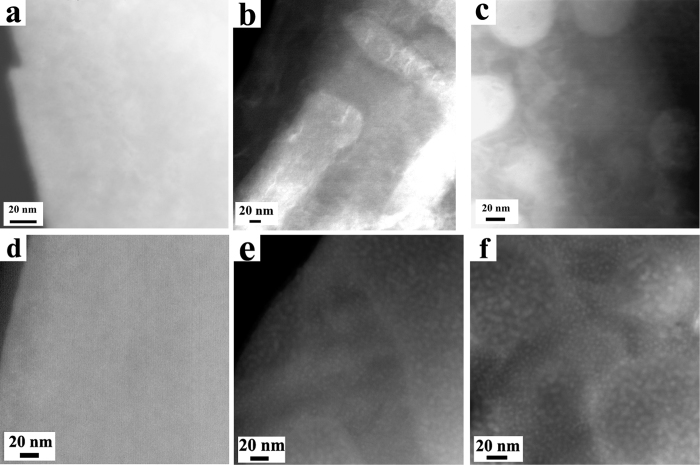
HAADF images of virgin (**a~c**) and as-irradiated (d**~f**) Al_*x*_CoCrFeNi HEAs. The ion irradiation was performed with 3 MeV Au ions at 1 × 10^16^ cm^−2^. (**a,c**) *x* = 0.1, (**b,e**) *x* = 0.75, (**c,f**) *x* = 1.5.

**Figure 4:**
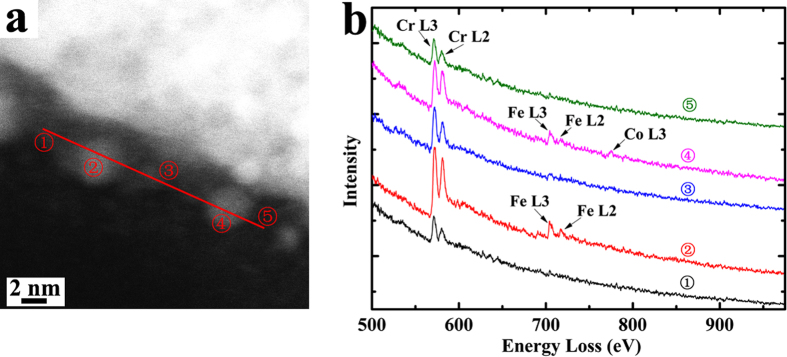
(**a**) HAADF image and (**b**) five representative EELS spectra of A2 phase in as-irradiated Al_1.5_CoCrFeNi.

From TEM results, it can be found that the most significant differences between the irradiation responses of three studied HEAs are the precipitation behavior. The single phase solid solution, Al_0.1_CoCrFeNi alloy, exhibits a great structural stability against ion irradiation, where precipitation was not observed in TEM characterization even at the highest fluence. In contrast, numerous precipitates can be observed in the irradiated multiphase Al_*x*_CoCrFeNi (*x* = 0.75 and 1.5) HEAs. Furthermore, the precipitates display different sizes and densities in the different phases. Figure 3 presents the HAADF-STEM images of virgin and as-irradiated Al_*x*_CoCrFeNi (3 MeV Au ions, 1 × 10^16^ cm^−2^), which clearly demonstrates the different precipitation behavior. Compared with matrix, all of these precipitates exhibit a brighter contrast. This suggests that precipitates contain a higher concentration of heavy elements, as compared with matrix, since the intensity in the HAADF image is approximately proportional to Z^2 ^18. Figure 4 shows the HAADF image and EELS line scan results of A2 phases in as-irradiated Al_1.5_CoCrFeNi (1 × 10^16^ cm^−2^, ~40 dpa). Five representative EELS spectra from the precipitates and matrix are given. It can be found that the precipitates contain a higher concentration of Fe than the matrix, which is consistent with the HAADF observations. For the Ni, Al-enriched B2 phases, Nano-EDX characterizations indicate the precipitates contain a higher concentration of Co, while the matrix contains a higher concentration of Al (not presented here). However, it should be noted that since the sizes of these precipitates are much smaller than the thickness of the TEM samples, both EELS and nano-EDX chemical characterizations have large quantitative error and can be only used to qualitatively measure the compositions of precipitates induced by ion irradiation.

**Figure 5:**
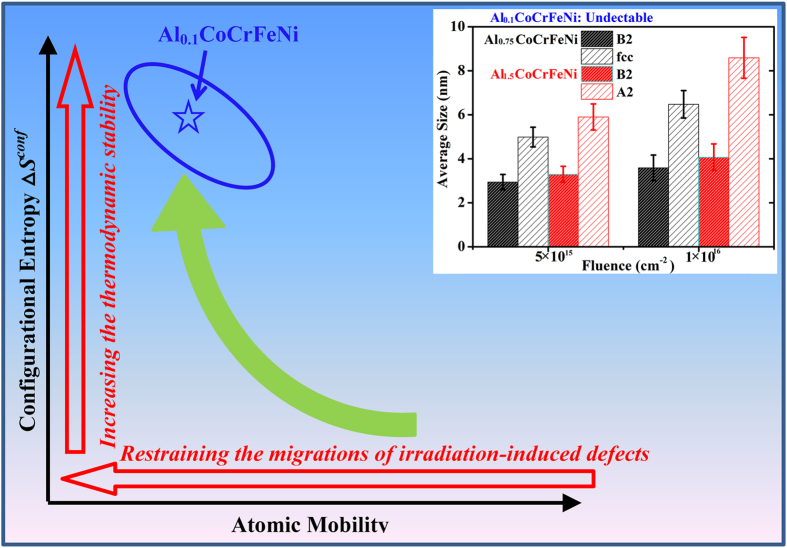
Schematic illustration of influences of configurational entropy and atomic mobility on the resistance of materials to precipitation under ion irradiation. The ellipse indicates the probable region where the materials are of great tolerance to precipitation and the inset shows the evolutions of precipitate sizes in different phases of as-irradiated Al_0.75_CoCrFeNi (black color) and Al_1.5_CoCrFeNi (red color).

The inset in Fig. 5 shows the precipitate sizes at 5 × 10^15^ cm^−2^ and 1 × 10^16^ cm^−2^; below 5 × 10^15^ cm^−2^ no precipitate can be observed in HAADF or BF images. The precipitates in the A2 phases of Al_1.5_CoCrFeNi have the largest size and the average precipitate sizes in the Cr, Fe-enriched fcc and A2 phases are obviously larger than those in the Al, Ni-enriched B2 phases for both HEAs, suggesting chemical composition plays a more important role in the precipitation behavior than structure in current study. The precipitates in the A2 phases of Al_1.5_CoCrFeNi exhibit a distinct growth with increasing fluence, which is mainly due to the precipitate coalescence, as shown in Fig. 3(f). In contrast, the growth of precipitates in the B2 phases is slow, indicating the precipitate sizes approach a saturation value. No precipitate was observed in as-irradiated Al_0.1_CoCrFeNi, indicating high phase stability or else any precipitate size or the composition difference between precipitates and matrix is too small to be detectable by TEM measurements.

## Discussion

In order to interpret the different precipitation behavior between single phase HEA and multiphase HEAs, we first summarize the discrepancies between both HEAs which may influence the precipitation behavior, and then give the explanation based on precipitation theory. It is well known that the high configurational entropies may stabilize solid solutions in equiatomic, multi-element HEAs. Many attractive properties of HEAs are attributed to the effects induced by the structure of multi-element solid solution, such as large lattice distortion. F. Otto *et al.*^19^ studied the microstructures of six equiatomic quinary alloys with analogous compositions and found that only one of the six alloys formed a single phase solid solution. Furthermore, these experimental results and the related thermodynamic analyses suggest that only the multi-element solid solutions, such as CoCrFeMnNi, have the true high configurational entropies, while for the multi-phase alloys exhibiting ordered phases or in which some kind of phase separation occurs, the configurational entropy, in fact, is not high. Therefore, these multi-phase alloys do not exhibit the characteristic properties and behavior induced by the structure of multi-element solid solution.

In current study, it was found that each phase in both multiphase HEAs consists of at least two or three major alloying elements. For example, Al_1.5_CoCrFeNi exhibits of a B2 and A2 duplex microstructure with the total concentration of Al, Co and Ni in B2 phases and Fe, Cr in A2 phases reaching 85.5 at. % and 84.7 at. %, respectively. It should be noted that no significant atomic diffusion and mixing between different phases can be observed after ion irradiation, suggesting the interaction between different phases and the effects of grain boundaries in the irradiation processes can be neglected. Therefore, it is assumed that each phase can be individually analyzed and be taken as corresponding binary or ternary concentrated alloys with minor alloying elements; their microstructural evolutions under ion irradiation is not influenced by other phases. For example the B2 phases in Al_1.5_CoCrFeNi can be taken as an Al-Co-Ni ternary alloy and A2 phases can be taken as a Fe-Cr binary alloy. Since there is no theoretical and experimental investigation about the precipitation behavior in concentrated alloys with principal elements *n* ≥ 3, it requires such assumption to simplify the model in order to interpret and compare the current experiment results.

If the effects of other minor constituents can be neglected, these compositions of different phases in multi-phase HEAs are far away from the solute composition regions where the solid solutions can be formed according to corresponding binary and ternary phase phase diagrams^15^,^20^. Therefore, the thermodynamic instability provides the driving force for the precipitation under ion irradiation, which is similar with the precipitation behavior of many immiscible binary systems^21^,^22^,^23^,^24^,^25^,^26^,^27^. Table 2 gives the chemical mixing enthalpies of different binary solid solutions^28^,^29^,^30^. It can be observed that the mixing enthalpy for Fe-Cr is very positive, which provides the thermodynamic driving force for the precipitation in A2 and fcc phases. The generation of excess vacancies or mobile interstitials from damage cascade events can enhance the kinetics of nucleation, and nucleation centers preferentially absorb the interstitials of Fe, promoting the growth of precipitates. In many Fe-Cr model and engineering materials with Cr concentrations above 10 at.%, ion irradiation can also induce or enhance the precipitation through homogeneous nucleation mechanism. However, the precipitates are generally enriched with Cr instead of Fe^21^,^27^,^31^. The difference is probably due to high content of Cr in A2 and fcc phases in the current study^31^,^32^,^33^.

For the B2 phases, both mixing enthalpies of Ni-Al and Co-Al are very negative. However, the mixing enthalpy of Ni-Co is the most positive between the five alloying components, which provides the thermodynamic drive for the precipitation of Co in the irradiation. Although the atomic number of Ni is larger than Co, the matrix contains a higher concentration of Al, which results in that the matrix exhibits a darker contrast. Moreover, based on the precipitation behavior in immiscible alloys under ion irradiation^34^, the smaller precipitate sizes for B2 phases, as shown in Fig. 5, may be induced by the lower atomic mobilities or the smaller thermodynamic driving force.

In contrast, the configurational entropy of single phase HEA Al_0.1_CoCrFeNi is much higher, which competes with the positive mixing enthalpy in the ion irradiation and reduces the thermodynamic driving force for precipitation. Furthermore, due to the high configurational entropy the thermodynamic stability of single phase Al_0.1_CoCrFeNi is increased with temperature increasing^10^, enhancing the stability of random solid solution during the evolution of high energy displacement cascades induced by ion irradiation. Another reason for the great resistance of single phase HEA to precipitation may be the sluggish atomic diffusion. The formation of precipitates in the ion irradiation is attributed to the fluxes of irradiation-induced defects, the defect fluxes towards various sinks result in the solute depletion or segregation at sinks. Many investigations^21^,^35^,^36^,^37^,^38^,^39^ have shown that atomic diffusion in multicomponent HEA is suppressed, resulting in a very low mobility. K.-Y. Tsai *et al.*^21^ studied the sluggish diffusion in an ideal-solution-like Co-Cr-Fe-Mn-Ni HEA and proposed that great fluctuation of lattice potential energy causes the significant atomic traps and blocks, leading to the high activation energies and low diffusion mobilities. The great fluctuation of lattice potential energy is attributed to the multi-component equiatomic solid solution microstructure, therefore only single phase HEAs are of the low atomic mobilities, leading to the great resistance to precipitation under ion irradiation.

As discussed above, the great structure stability of single phase HEA Al_0.1_CoCrFeNi under ion irradiation is attributed to the high configurational entropy and low atomic mobility, which reduces the thermodynamic driving force and kinetically restrains the formation of precipitate, respectively. It should be noted that the two factors are not independent, both are resulted from the multicomponent equiatomic solid solution structure. The schematic illustration of dependence of the resistance to precipitation on configurational entropy and atomic mobility is shown in Fig. 5. The blue ellipse indicates the probable region where the materials are of great tolerance to precipitation, some other single phase HEA, such as CoCrFeNiMn, may also locate in this region. In fact, the effects of high configurational entropy on irradiation tolerances may be not only limited to precipitation. Recently, D S. Aidhy *et al.* studied the irradiation-induced point defect evolution in Ni, NiFe and NiCr alloys combining atomistic simulations and irradiation experiments and found that the kinetics of defect formation in NiFe and NiCr alloys is much slower than pure Ni, resulting in the smaller defect sizes in both binary alloys, which is mainly due to the higher defect migration barriers and extended defect formation energies for binary alloys^13^. This suggests the formation and growth of irradiation-induced defects are restrained in multicomponent equiatomic solid solution and the single phase HEAs are of great potential for using as nuclear materials. It should be noted that recently, K. Jin *et al.*^40^ observed the irradiation-induced voids and its related volume swelling in various multi-component alloys at 500 °C and N.A.P. Kiran Kumar *et al.*^41^ found the irradiation-induced segregation in grain boundaries in FeNiMnCr HEA irradiated at high temperatures. These experimental phenomena are not observed in current study, indicating the irradiation responses of HEA at high temperatures are drastically different from that of room temperature. Further systematic studies on the irradiation responses of HEAs at elevated temperatures are required for their applications in advanced nuclear systems.

## Conclusions

In summary, three multicomponent HEAs Al_*x*_CoCrFeNi (*x* = 0.1, 0.75 and 1.5) were irradiated with 3 MeV Au ions. It was found that both multiphase HEAs exhibit significant precipitation following irradiation. In contrast, the single phase HEA Al_0.1_CoCrFeNi exhibits a great phase stability against ion irradiation, no precipitate can be found even at ~43 dpa. The great structure stability of single phase HEA against precipitation under ion irradiation is attributed to the high configurational entropy and low atomic mobility, which reduces the thermodynamic driving force and kinetically restrains the formation of precipitate, respectively. For multiphase HEAs, the formation of ordered phases and phase separation reduce their configurational entropies, resulting in that they behave like conventional binary and ternary alloys under irradiation. This study verified the great structure stability of single phase HEA under ion irradiation and demonstrated the effects high configurational entropy on the irradiation tolerance, which provides an important implication for searching for HEAs with higher irradiation tolerances.

## Methods

Alloy ingots with a nominal composition of Al_*x*_CoCrFeNi (*x* = 0.1, 0.75 and 1.5) were synthesized by arc-melting a mixture of pure metals (purity >99.6 wt.%) in a Ti-gettered high-purity argon atmosphere. These ingots were remelted at least four times to ensure chemical homogeneity. Melted alloys were eventually drop-cast into a mold Φ 5 × 70 mm^3^. The solidified ingots were cut into thin pieces 1 mm thick and one side polished. The sample densities for *x* = 0.1, 0.75 and 1.5, which were measured by Archimedes method, are 8.1, 7.4 and 6.9 g·cm^−3^, respectively. The microstructures of as-prepared Al_*x*_CoCrFeNi HEAs (*x* = 0.1, 0.75 and 1.5) are single fcc, fcc plus ordered bcc (B2) and B2 plus disordered bcc (A2), respectively. The XRD and SEM characterization results are given in Supplementary Figs S2 and S3, the detailed microstructure characterizations can be also found in our previous study^14^.

The microstructure and compositions of as-prepared Al_*x*_CoCrFeNi HEAs were first characterized by XRD (Philips X Pert Pro), SEM (FEI, Nano430) and TEM (FEI, Tecnai F30). TEM samples were prepared by mechanical polishing to approximately 100 μm thickness, followed by dual jet polishing in an ethanol solution containing 5% HClO_4_. To investigate the irradiation responses of the three HEAs, we directly irradiated TEM foils of HEA samples using 3 MeV Au ions at room temperature with fluences ranging from 1 × 10^14^ to 1 × 10^16^ cm^−2^, the ion flux was kept at ~3 × 10^11^ cm^−2^ s^−1^. The irradiation of 3 MeV Au was performed at a 2 × 1.7 MeV tandem accelerator facility. The corresponding ion range and displacements per atom (dpa) were calculated using SRIM 2008 with simple Kinchin-Pease method, the displacement energies for different atoms are all set as 40 eV^40^,^41^,^42^. The thicknesses of electron-transparent regions where the high-resolution observations can be performed, was estimated to be less than 100 nm. Therefore, the irradiation doses are taken as the average dpa within the depth of 100 nm. The irradiation fluences and corresponding dpa for the three studied HEAs are summarized in Supplementary Tab. S1 and the depth profiles of dpa and Au concentration for 3 MeV Au ions in Al_0.1_CoCrFeNi at 1 × 10^16^ cm^−2^ are shown in Supplementary Fig. S4 as an example.

The chemical compositions of virginal and as-irradiated samples were characterized by TEM-EDX under STEM nano-probe condition with a beam size of about 1 nm. In order to clarify the microstructural and compositional differences between matrix and irradiation-induced precipitates, a 200 kV aberration-corrected TEM (JEM-ARM200F) was used to measure the electron energy loss spectroscopy (EELS) and observe atomic-level high-angle annular dark field (HAADF) images.

## Additional Information

**How to cite this article**: Yang, T. *et al.* Precipitation behavior of AlxCoCrFeNi high entropy alloys under ion irradiation. *Sci. Rep.* **6**, 32146; doi: 10.1038/srep32146 (2016).

## Supplementary Material

Supplementary Information

## Figures and Tables

**Table 1:** Microstructures and chemical compositions (at.%) of virginal and as-irradiated Al_*x*_CoCrFeNi (*x* = 0.1, 0.75 and 1.5) HEAs.

Alloy	Microstructure	Al	Co	Cr	Fe	Ni
Al_0.1_CoCrFeNi (single fcc)	fcc	1.7 ± 0.7	25.6 ± 1.3	22.5 ± 2.3	25.3 ± 1.2	24.9 ± 2.2
	fcc (as-irradiated)	2.5 ± 0.9	22.9 ± 1.2	25.9 ± 1.8	24.2 ± 1.7	24.5 ± 2.2
Al_0.75_CoCrFeNi (fcc+B2)	fcc	6.2 ± 0.3	24.4 ± 2.6	26.1 ± 3.1	26.1 ± 3.0	17.0 ± 3.6
	fcc (as-irradiated)	4.6 ± 1.5	24.4 ± 1.5	27.4 ± 1.9	28.5 ± 1.3	15.1 ± 2.1
	B2	32.3 ± 2.1	17.9 ± 2.1	4.8 ± 0.8	11.5 ± 1.4	30.6 ± 5.5
	B2 (as-irradiated)	33.7 ± 4.2	17.6 ± 1.3	5.3 ± 2.6	11.7 ± 1.5	31.7 ± 2.0
Al_1.5_CoCrFeNi (A2+B2)	A2	5.0 ± 1.0	8.8 ± 0.6	52.9 ± 4.0	31.8 ± 4.8	1.1 ± 0.8
	A2 (as-irradiated)	2.1 ± 2.5	7.6 ± 3.3	56.4 ± 5.0	32.8 ± 3.2	1.0 ± 1.4
	B2	40.2 ± 2.6	21.3 ± 2.1	3.2 ± 1.1	11.2 ± 0.8	24 ± 5.3
	B2 (as-irradiated)	37.1 ± 3.0	22.5 ± 1.6	4.1 ± 2.5	13.5 ± 1.9	22.6 ± 2.4

**Table 2:** Enthalpies of mixing (kJ·mol^−1^) of binary systems containing the elements in Al_*x*_CoCrFeNi.

Binary System	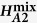	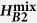	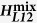
Al-Co	−24	−58	−34
Al-Cr	−6	−14	−10
Al-Fe	−10	−36	−28
Al-Ni	−30	−66	−41
Co-Cr	8	6	3
Co-Fe	13	9	/
Co-Ni	17	/	/
Cr-Fe	13	6	5
Cr-Ni	3	5	1
Fe-Ni	12	8	/

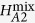
, 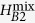 and 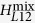 represent the mixing enthalpies of forming A2, B2 and L12 structure, respectively^28^,^29^,^30^.
