# Supplementary material for: Precipitation behavior of AlxCoCrFeNi high entropy alloys under ion irradiation
Source: Sci Rep. 2016 Aug 26;6:32146. doi: 10.1038/srep32146 (PMC4999872; doi:10.1038/srep32146)
Supplement: Supplementary Information [file srep32146-s1.doc]

**Precipitation behavior of** **Al*_x_*CoCrFeNi high entropy alloys under ion irradiation**

**Tengfei Yang ^1^, Songqin Xia ^2^, Shi** **Liu ^2^, Chenxu Wang ^1^, Shaoshuai Liu ^1^, Yuan Fang ^1^, Yong Zhang ^2^, Jianming Xue ^1^, Sha Yan ^1^, Yugang Wang ^1,*^**

^1^*State Key Laboratory of Nuclear Physics and Technology, Center for Applied Physics and Technology, Peking University, Beijing 100871, People’s Republic of China*

^2^*State Key Laboratory for Advanced Metals and Materials, University of Science and Technology Beijing, Beijing 100083, China*

* Corresponding author: [ygwang@pku.edu.cn](mailto:ygwang@pku.edu.cn) Tel: + 86-10-62755406

**Figure S1**

**
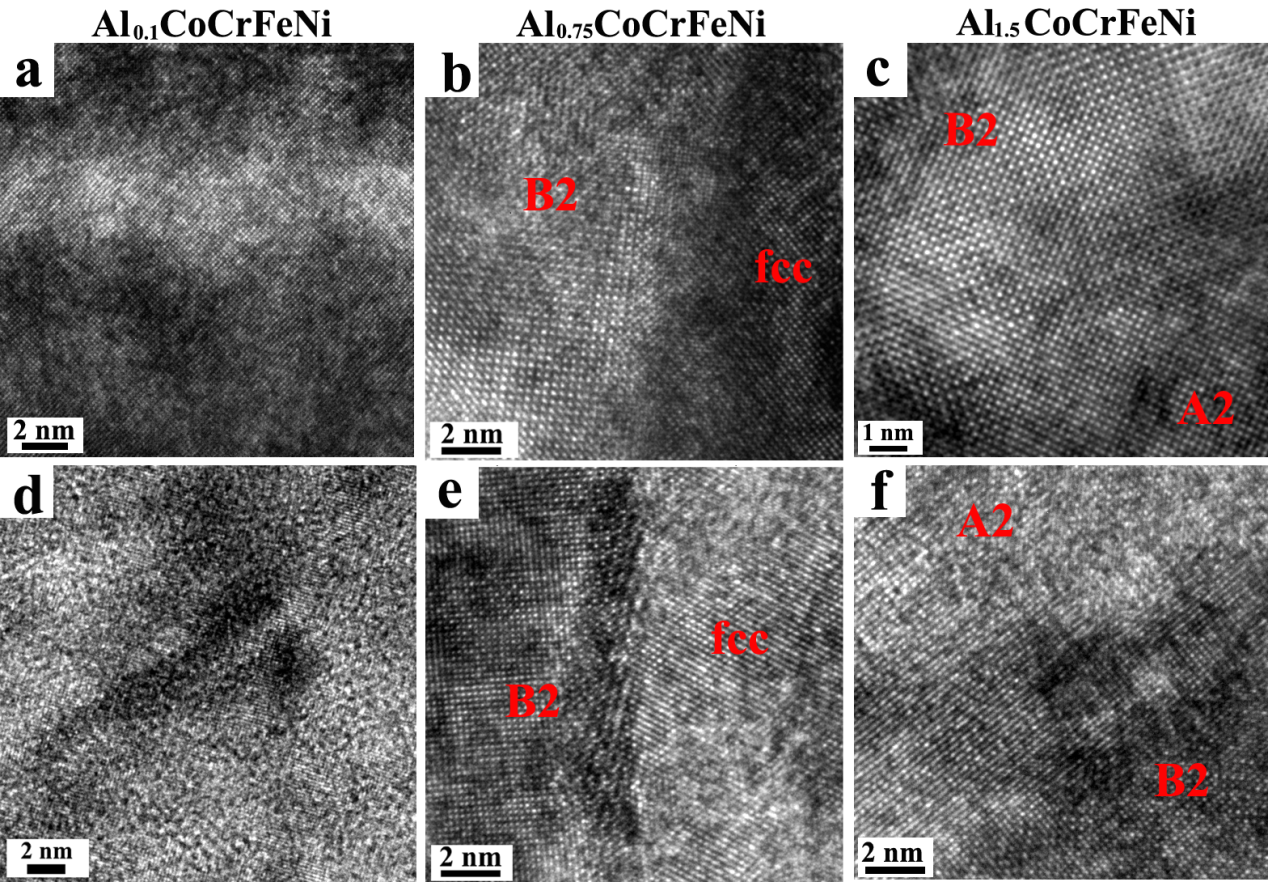
**

**Figure S1. HRTEM images of (a)~(c) virginal Al*_x_*CoCrFeNi HEAs and (d)~(f) Al*_x_*CoCrFeNi HEAs irradiated with 3 MeV Au ions at 1×10^16^ cm^-2^. (a), (d) *x*=0.1; (b), (e) *x*=0.75; (c), (f) *x*=1.5. All images are taken with electron beam along [100] zone axis. For *x*=0.75 and 1.5, the HRTEM images are taken at grain boundaries to exhibit the irradiation-induced structural damage in both phases.**

**Figure S2**

**
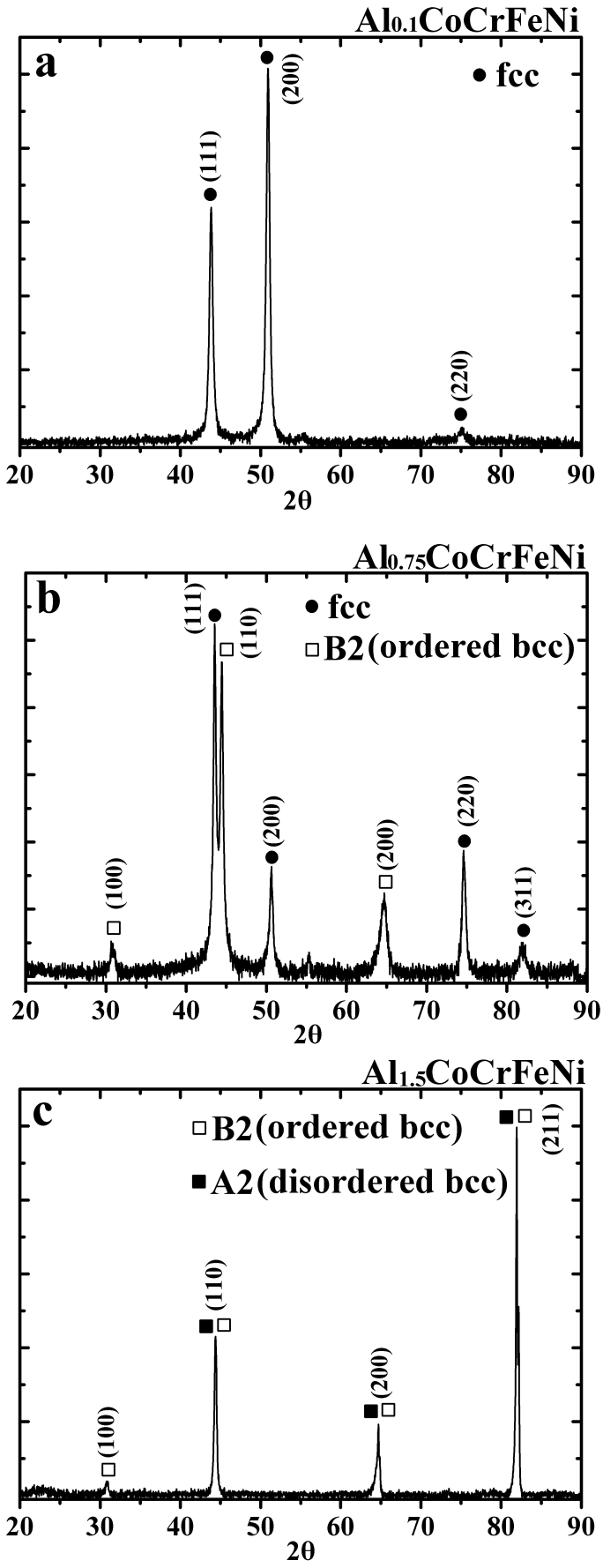
**

**Figure S2. XRD patterns of virgin (a) Al_0.1_CoCrFeNi, (b) Al_0.75_CoCrFeNi and (c) Al_1.5_CoCrFeNi.**

**Figure S3**

**
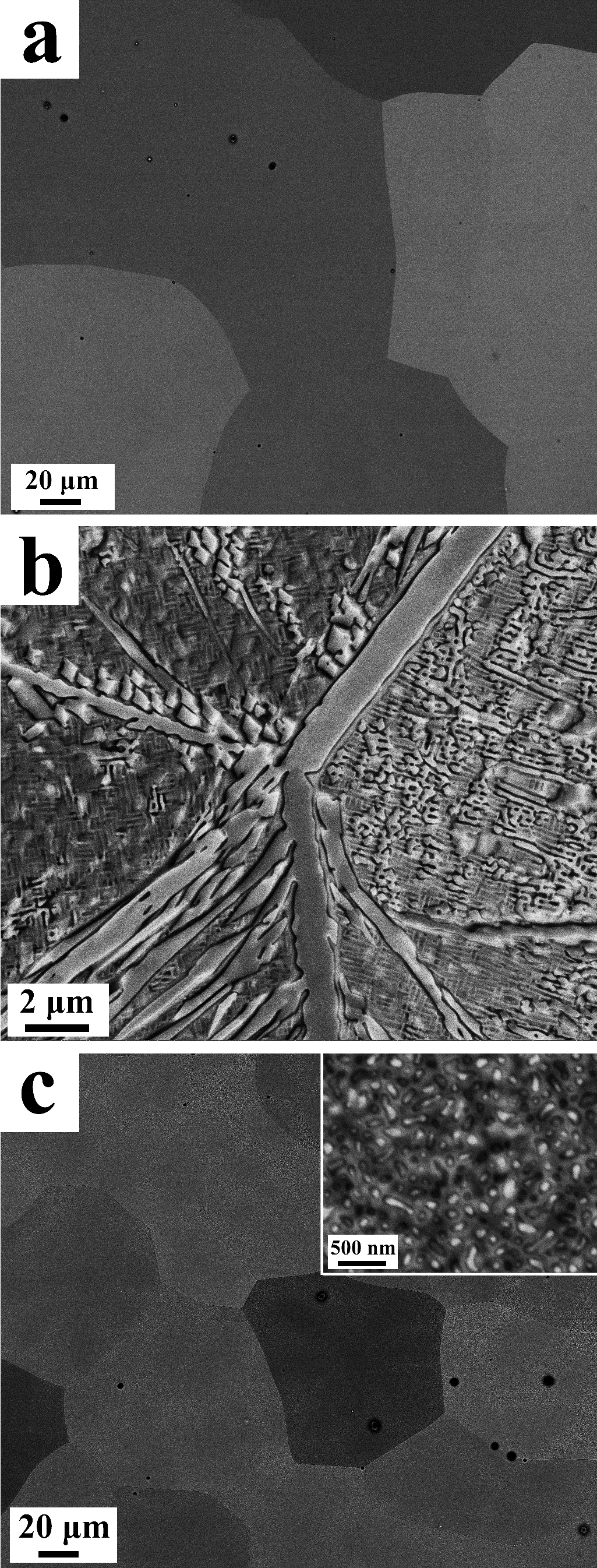
**

**Figure S3. SEM images of virgin (a) Al_0.1_CoCrFeNi, (b) Al_0.75_CoCrFeNi and (c) Al_1.5_CoCrFeNi. The inset in (c) shows that numerous precipitates distributing in the matrix.**

**Figure S4**

**
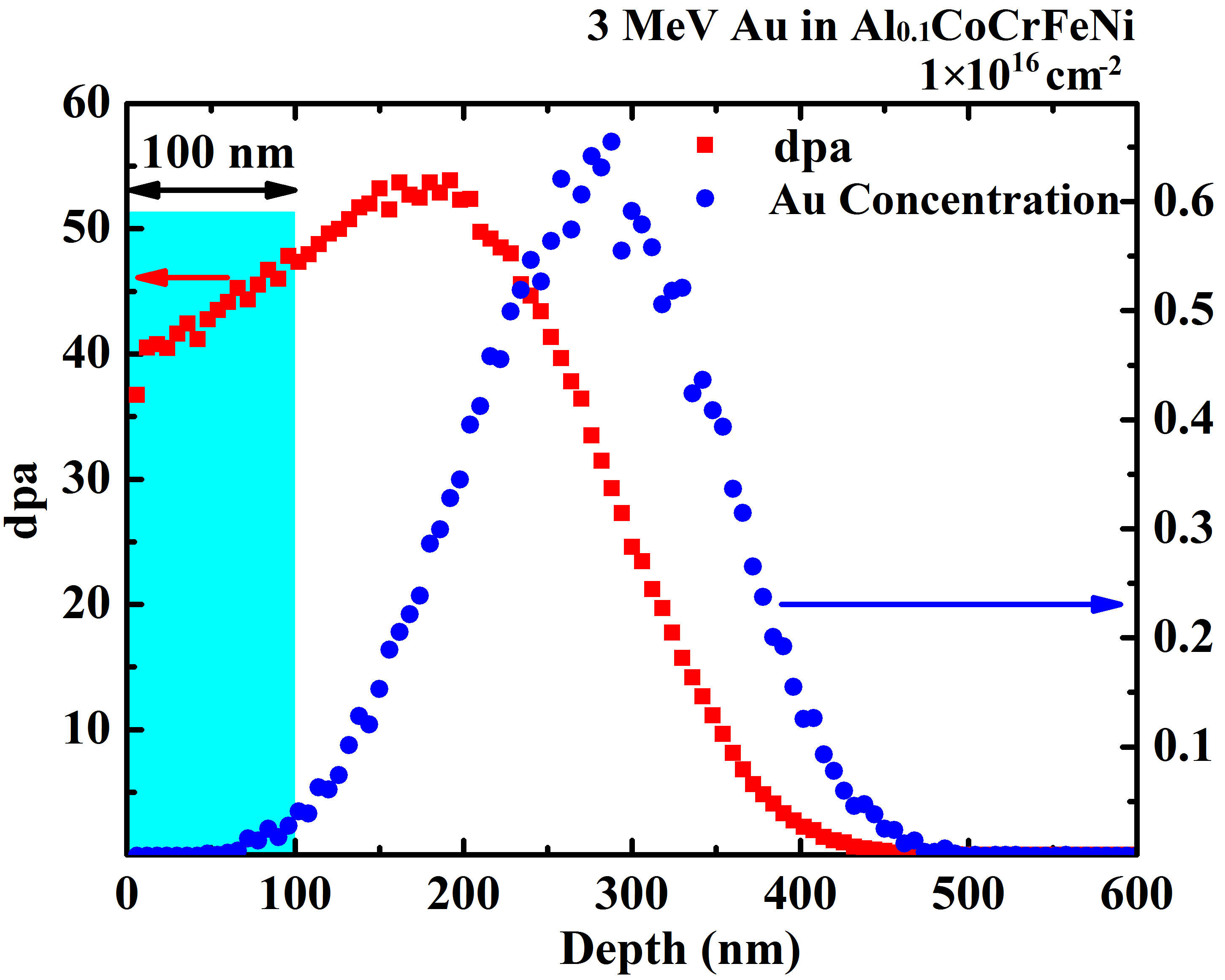
**

**Figure S4. Depth profiles of damage (dpa) and deposited Au concentration (at. %) for Al_0.1_CoCrFeNi irradiated with 3 MeV Au ions at 1×10^16^ cm^-2^. The dpa and Au concentration for irradiated TEM foils are estimated according to corresponding average values within the depth region of 0 to 100 nm.**

| Alloy | Fluence (ion·cm^-2^) | dpa |
| --- | --- | --- |
| Al_0.1_CoCrFeNi | 1×10^14^ ~ 1×10^16^ | 0.43 ~ 43 |
| Al_0.75_CoCrFeNi | 1×10^14^ ~ 1×10^16^ | 0.40 ~ 40 |
| Al_1.5_CoCrFeNi | 1×10^14^ ~ 1×10^16^ | 0.37 ~ 37 |

**Table S1 The irradiation fluences and corresponding dpa for the three studied HEAs irradiated with 3 MeV Au ions.**
